# Anticonvulsant Potential of the Essential Oil of *Croton Heliotropiifolius* Kunth: In Vivo and In Silico Approach

**DOI:** 10.1021/acsomega.5c04107

**Published:** 2026-02-04

**Authors:** Maria Elane S. da Cunha, Angélica L. Soares, Esdras M. S. Lima, Francisco A. S. Filho, Ricardo M. Ramos, Rosemarie B. Marques, Francisco Das C. P. de Andrade, Anderson N. Mendes, Evandro Paulo S. Martins

**Affiliations:** † Postgraduate Program in Chemistry, State University of Piaui, Teresina, Piauí 64002-150, Brazil; ‡ 316703 State University of Piauí, Campus Antonio Giovanne Alves de Sousa, Piripiri, Piauí 64260-000, Brazil; § State University of Piauí, FACIME, Teresina, Piauí 64002-150, Brazil; ∥ State University of Piauí, Campus Professor Alexandre Alves de Oliveira, Parnaíba, Piauí 64202-262, Brazil; ⊥ Laboratory of Molecular Biology and Epidemiology (LABME), Federal Institute of Education, 119529 Science and Technology of Piauí, Teresina, Piauí 64002-150, Brazil; # Biotechnology and Biodiversity Research Center, State University of Piauí, Teresina, Piauí 64002-150, Brazil; ∇ Laboratory of Innovation in Science and Technology − LACITEC, Department of Biophysics and Physiology, 67823 Federal University of Piauí, Teresina, Piauí 64049-550, Brazil; ○ Department of Biophysics and Physiology, Federal University of Piauí, Teresina 64049-550, Brazil

## Abstract

Epilepsy is a chronic condition that significantly impacts the quality of life of many individuals, underscoring the urgent need for the identification of safe and effective anticonvulsant agents. In this context, medicinal plants have emerged as a promising source of bioactive compounds for treating epilepsy. This study involved an in vivo and in silico investigation of the anticonvulsant activity of the essential oil from the leaves of *Croton heliotropiifolius* Kunth (OCH). In vivo experiments revealed that the essential oil promoted a significant increase in seizure latency and survival rate in animals treated with OCH at a dose of 200 mg/kg, indicating an anticonvulsant effect. To understand the possible receptors and sites of action of the compounds in the oil, we performed a molecular docking study with GABA_A_ and NMDA receptors. Additionally, we calculated the electronic properties of the phytoconstituents at the B3LYP/6–311++G­(d,p)/SMD level. The results of the molecular docking studies revealed that the sesquiterpenes α-bulnesene, δ-cadinene, and β-bourbonene, which are present in OCH, have a high affinity for the GABA_A_ receptor, with binding energies ranging from −10.0 to −9.1 kcal/mol. These compounds primarily interact with the receptors through hydrophobic forces, highlighting the importance of interaction with Phe77 of the γ2^–^(E) subunit of GABA_A_. Docking analysis of NMDA revealed a higher affinity for the sesquiterpene guaiadiene, with a binding energy of −8.0 kcal/mol. Molecular dynamics simulations indicate that the α-bulnesene–GABA_A_ and guaiadiene–NMDA complexes remained stable over 100 ns. DFT analysis revealed that the most promising ligands are more stable and have moderate to strong electrophilicity. This research provides valuable insights for the identification of new molecules in the development of herbal medicines for the treatment of epilepsy, suggesting that the anticonvulsant effect of OCH may be related to the modulation of the GABA_A_ receptor or NMDA.

## Graphical abstract

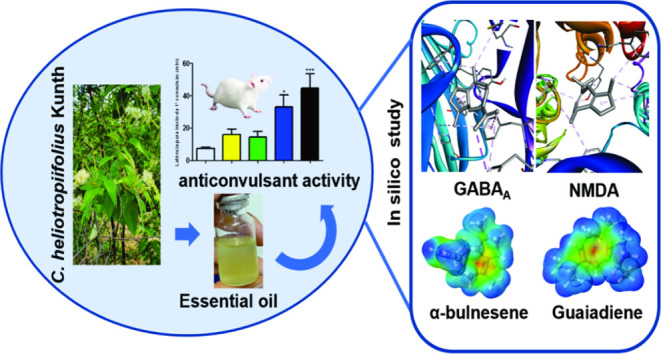

## 1 Introduction

Epilepsy is a neurological disorder characterized by an excitatory or inhibitory imbalance that occurs spontaneously and recurrently in the central nervous system and affects more than 75 million people worldwide.^1^ In the study of the pathophysiology of epilepsy, seizures involve various neurotransmitter systems such as GABAergic,^2^ glutamatergic,^3^ serotoninergic,^4^ dopaminergic,^5^ etc. They are therefore the target of many drugs and herbal medicines to combat epilepsy.

Type-A γ-aminobutyric acid receptors (GABA_A_) is one of the major inhibitory receptors in brain synapses^6^ and is targeted by many drugs that act at different binding sites, such as benzodiazepines (BDZs), which act as modulators allosteric positive at the GABA_A_/BDZ binding site, which enhances the activity of the inhibitory neurotransmitter GABA, responsible for reducing neuronal hyperexcitability and the subsequent development of convulsive or epileptic seizures,^7^ which justifies the choice of GABA_A_ as an important molecular target. Other receptors that have been implicated in epilepsy are the extrasynaptic *N*-methyl-d-aspartate (NMDA) receptors, composed primarily of the GluN1 and GluN2B subunits, which have been shown to play a critical role in preventing neuronal death associated with glutamate toxicity.^8^ Furthermore, recent studies have identified the GluN2B subunit as being one of the main ones associated with the pathogenesis of recurrent seizures,^9^ thereby reinforcing its status as a promising target.

4-[(1*R*,2*S*)-2-(4-Benzylpiperidin-1-yl)-1-hydroxypropyl]­phenol (Ifenprodil) is a selective antagonist of the NMDA receptor containing the GluN2B subunit with proven antiepileptic properties. Administering it significantly reduces NMDA-mediated synaptic currents in the hippocampus and temporal cortex.^10^ In addition, ifenprodil demonstrated selective antiepileptic effects in five patients with refractory epilepsy associated with cortical developmental malformations.^11^

Treatment of seizures and epileptic convulsions is usually with synthetic drugs such as diazepam, valproate sodium, phenytoin and carbamazepine.^12,13^ However, in approximately 25–45% of cases, the medications used in treatment are insufficient to control seizures and do not produce an adequate response.^14^ In addition, they may have adverse effects such as sedation, depression, insomnia and fatigue.^15^ A promising approach to finding new anticonvulsant drugs and improving epilepsy therapy is to explore natural compounds from plants used in folk medicine.

Traditional communities use medicinal plants as home remedies for their therapeutic properties and as raw materials for the production of herbal medicines and other drugs.^16^ There are many products that can be obtained from medicinal plants, including essential oils (EOs), which are volatile, odorous substances that are immiscible or very slightly miscible in water and act in biological functions that are important for plant survival,^17^ related to defense mechanisms such as protection against microorganisms, insects and animals.^18^

The genus *Croton* sp. belongs to the family *Euphorbiaceae*, which includes about 300 genera and 8000 species found all over the world, especially in America and Africa. This group is found in different habitats, depending on the size of the trees. They have monogamous flowers and the fruits usually have capsules with three parts, each containing an oily seed.^19^

Studies on the composition of the essential oil from the leaves of the species *Croton heliotropiifolius* Kunth (OCH) revealed a mixture of monoterpenes (63.79%) and sesquiterpenes (32.98%) as chemical constituents.^20^ Monoterpenes have muscle relaxant, antimicrobial, antispasmodic, antidepressant, anti-inflammatory, anxiolytic and anticonvulsant properties.^21,22^

Marques and collaborators conducted an in vivo and in silico study to evaluate the pharmacokinetic and toxicological properties of the chemical constituents present in OCH. The in silico analysis showed that all compounds exhibited high oral absorption, moderate cellular permeability and high permeability across the blood-brain barrier. Toxicity tests indicated that the constituents were of low to moderate toxicity. Furthermore, the mutagenicity test showed that there were no significant changes in the number of micronuclei. These results suggest the potential of the essential oil of this plant as a candidate for the development of new drugs.^23^

In this work, we performed an in vivo and in silico investigation of the anticonvulsant activity of the essential oil from the leaves of the plant *C. heliotropiifolius* Kunth. The molecular docking study of the 33 constituents of OCH with the GABA_A_ and NMDA receptors was performed to evaluate the affinity and the nature of the intermolecular interactions. Molecular dynamics simulations were performed in order to evaluate the stability of the ligand–receptor complexes. In addition, the electronic properties of the ligands were investigated using Density Functional Theory (DFT). The frontier molecular orbitals, the electrostatic potential map and some chemical reactivity descriptors often correlated with biological activities were calculated.^24–26^

## 2 Methodology

### 2.1 Ethical Procedures

The work followed the guidelines recommended by law 11.794 of the National Council for the Control of Animal Experimentation. The project was submitted to the Ethics Committee for the Use of Animals (CEUA) of the State University of Piauí and approved under protocol number 006201/2022-10. The activity of access to Associated Traditional Knowledge, under the terms required by the National System for the Management of Genetic Heritage and Associated Traditional Knowledge—SisGen, in compliance with the provisions of Law no. 13,123/2015 and its regulations, was registered under number A8B513F.

### 2.2 Experimental Protocol

High doses of pilocarpine induce status epilepticus or status mal epilepticus (SMA), a prolonged seizure that causes brain damage similar to an epileptogenic condition in humans.^27^ Male *Mus musculus* mice (25–30 g), in numbers of 6 animals per group (*n* = 6) from the Bioterium of the State University of Piauí were used due to availability in the vivarium. The animals were divided into 5 groups: Negative control group: received 0.9% saline solution orally (v.o.) at a dose of 0.1 mL/10 g; Positive control group: received diazepam 4 mg/kg intraperitoneally (i.p.); Test groups: received essential oil from the leaves of*C. heliotropiifolius* at doses of 50, 100, and 200 mg/kg (v.o.)

The essential oil from the leaves of*C. heliotropiifolius* was purchased from the Chemistry Laboratory, Campus Prof. Alexandre Alves de Oliveira, UESPI, Parnaíba-PI, Brazil.

All animals were treated with saline solution or oil for 1 h or diazepam for 30 min before induction of seizures. After the 1 h or 30 min of the aforementioned treatments, methylscopolamine 1 mg/kg was administered (to all animals). After 30 min, pilocarpine 400 mg/kg (i.p.) was administered to all groups. The purpose of administering methylscopolamine was to attenuate peripheral effects caused by pilocarpine injection, such as hypersecretion.

To compare the severity of seizures between groups after administration of this chemical agent, the following parameters were observed: latency to onset of seizures and latency to death. Each animal was observed for 1 h. The latency to onset of seizures and the time until death of the animal were recorded in minutes.

### 2.3 Statistical Analysis of Data

The results were evaluated using the ANOVA method, followed by Tukey’s posttest, using the GraphPad Prism 5.0 statistical program. The significance level was 95%.

### 2.4 Molecular Docking

#### 2.4.1 Preparation of Ligands

The three-dimensional structures of the 33 ligands and reference drugs (diazepam, clonazepam and ifenprodil) were obtained from the PubChem platform (https://pubchem.ncbi.nlm.nih.gov/) and their structures were optimized using DFT calculations in aqueous solution using the B3LYP functional^28^ and the 6–311++G­(d,p) basis set,^29^ using the Orca 5.0.3 software.^30^ The solvent effect was simulated using the SMD implicit model.^31^ The optimized structures of the compounds were then converted into PDB format using the Avogadro program (version 1.2.0.) and then protonated under physiological conditions (pH = 7.4) using the OpenBabel (version 3.1.0). The Gasteiger atomic charges and polar hydrogens were then added to the structures, with the nonpolar hydrogens being suppressed. Finally, the files were converted to PDBQT format using AutoDock Tools (version 1.2.0).

#### 2.4.2 Preparation of Proteins

The α1β2γ2 subtype of the human GABA_A_ receptor, obtained by electron microscopy and complexed with the neurotransmitters GABA and diazepam (PDB ID: 6X3X), and the crystallographic structure of the GluN1 and GluN2B subunits of the NMDA receptor, complexed with the negative allosteric modulator ifenprodil (PDB ID: 5EWJ),^32^ were obtained from the RCSB database (http://www.rcsb.org). The UCSF ChimeraX software^33^ was used to remove the ligands and water molecules and to isolate the binding sites.

An initial inspection of the 6X3X and 5EWJ structures was carried out to identify atomic overlap and missing residues. Next, comparative modeling was employed using the MODELER 10.4 ^34^ software, which uses the spatial constraints method to generate 3D structures and reconstruct gaps in the original coordinates. Due to the high identity and sequence coverage obtained in the PDB, the 6X3X and 5EWJ structures themselves were used as templates. The target sequences were aligned to the templates using EMBOSS Needle. The new 3D models were then generated using MODELER and the models with the best DOPE scores were selected. Conformational quality was validated by analyzing the psi (ψ) and phi (φ) torsional angles in Ramachandran plots.^35^ Finally, the refined structures were aligned with the originals in PyMOL for comparison.

The GABA_A_ binding sites selected for the study include the classic benzodiazepine site, located at the extracellular interface α1^+^(D)/γ2^–^(E) (site 1), and three diazepam binding sites identified in the intracellular domain, located at the interfaces: β2^+^(C)/α1^–^(D) (site 2), β2^+^(A)/α1^–^(B) (site 3) and β2^–^(A)/γ2^+^(E) (site 4), Figure 1.^36^

**Figure 1:**
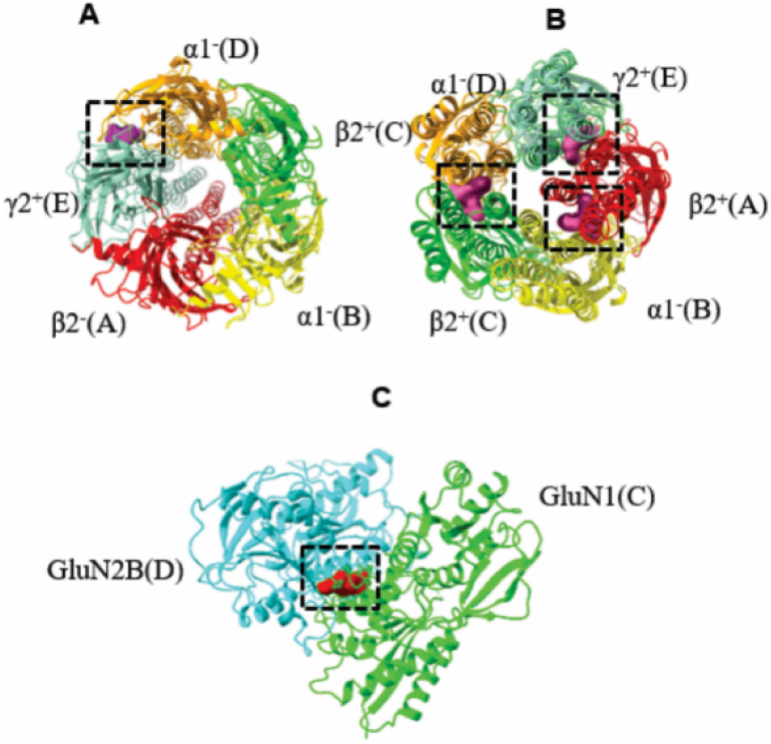
Diazepam binding sites on the GABA_A_ receptor: (a) An extracellular domain binding site for diazepam at the α1+(D)/γ2-(E) interface. (b) Three additional diazepam binding sites in the transmembrane domain at the β2+(C)/α1-(D), β2+(A)/α1-(B) and β2-(A)/γ2+(E) interfaces and (c) ifenprodil site on the NMDA receptor.

Additionally, the binding site of the ifenprodil on the NMDA receptor was investigated. This site is located at the GluN1­(C)-GluN2B­(D) interface (**site 5**) of the NMDA receptor (Figure 1C).

Once the sites were selected, the structures were protonated at physiological conditions (pH = 7.4) using the PDB2PQR platform (https://pdb2pqr.readthedocs.io/en/latest/). Subsequently, the Gasteiger atomic charges^37^ and polar hydrogens were added and nonpolar hydrogens were suppressed. The structures were then converted to PDBQT format using AutoDock Tools.

#### 2.4.3 Molecular Docking Protocol

Molecular docking was performed using the Autodock Vina software^38^ (version 1.1.2), following the methodology of Barros and coworkers.^39^ The size of the grid box was set to 22.5 Å for each axis. The grid box was centered on the coordinates of the oxygen atoms of residues SER D:205, ILE D:228, GLN A:224 and ILE B:228 identified at binding sites 1, 2, 3, and 4 of the GABA_A_, respectively. The same procedure was applied to the NMDA receptor, with residue C:SER132 (corresponding to site 5) selected as the center of the docking box (see Table S1).

The number of modes (poses) was set to 50 and the completeness to 24 to ensure a comprehensive analysis. The docking simulations were conducted using the rigid structure of the proteins and the flexible ones of the ligands.

Redocking was performed with the ligands diazepam (DZP) and ifenprodil in their respective binding sites. The redocking calculations, the poses of the ligand-protein complexes, and the intermolecular interactions were visualized using the Discovery Studio.^40^

### 2.5 DFT Study

The electronic properties of the ligands were calculated at the same theoretical level as the geometry optimization. Molecular electrostatic potential (MEP) maps were generated in the program Avogadro (version 1.2.0) with an isosurface value of 0.002 au and visualized in the Jmol program (version 14) to identify the nucleophilic and electrophilic reactivity regions of the molecules. The HOMO and LUMO frontier molecular orbitals were calculated and their energies were used to obtain some global chemical reactivity descriptors, such as ionization potential (*I*), electronic affinity (*A*),^41^ electronic chemical potential (μ),^42^ hardness (η),^43^ electronegativity (χ),^44^ electrophilicity (ω)^45^ and HOMO–LUMO energy gap.

### 2.6 Molecular Dynamics Simulations

Molecular dynamics simulations were performed using GROMACS 2024.2 (GPU)^46^ with the best docking poses for GABA_A_ (site 1) and NMDA (site 5). The topologies were generated using CHARMM-GUI^47,48^ and the simulations were conducted in triplicate.

Solvation was performed using the TIP3 model in a cubic box, with a distance ≥10 Å between the protein and the box edges. This was followed by neutralization with Na^+^ and Cl^–^ ions. Topologies were generated using CHARMM36 for proteins and CGenFF for ligands.

Energy minimization (steepest descent) was performed until a maximum force of 100 kJ/(mol·nm) was reached. Equilibration occurred in both NVT (1 ns) and NPT (1 ns) without restrictions. Production (100 ns) was performed in NPT with a 2-fs time step and H-bond restriction using the LINCS algorithm. Temperature (310 K) and pressure (1 atm) were controlled using V-rescale (τ = 0.1 ps) and C-rescale (τ = 5 ps) couplings, respectively. Electrostatic interactions were treated using PME with periodic boundary conditions in all directions.^49,50^

The root-mean-square deviation (RMSD), root-mean-square fluctuation (RMSF), radius of gyration (*R*_g) and solvent-accessible surface area (SASA) of the protein–ligand systems were determined using the gmx module, and Xmgrace software was used to obtain 2D graphs of these parameters.

Free binding energies were calculated using the molecular mechanics/Poisson–Boltzmann surface area (MM-PBSA) method. Van der Waals interactions, electrostatic interactions, potential energy and polar and nonpolar solvation were calculated using the gmx_MMPBSA tool based on AMBER’s MMPBSA.py to perform free energy calculations of the final state with GROMACS files.^51^ The VMD and PyMOL visualization tools were used to examine the trajectory and details of the interactions.

## 3 Results and Discussion

### 3.1 Anticonvulsant Activity In Vivo

After treatment with vehicle (0.1 mL/10 g of animal, v.o.), diazepam (4 mg/kg, i.p.) and *C. heliotropiifolius* leaf oil (50, 100 and 200 mg/kg, v.o.), there was an increase in the latency of the first seizure in the group that received OCH at a dose of 200 mg/kg (33.00 ± 18.47, *p < 0.05, *p < 0.05), there was an increase in first seizure latency in the group of animals treated with OCH at a dose of 200 mg/kg (33.00 ± 18.47, *p < 0.05) and diazepam (44.85 ± 21.27, ***p < 0.001) compared to the negative control (7.43 ± 1.74). (Figure 2).

**Figure 2:**
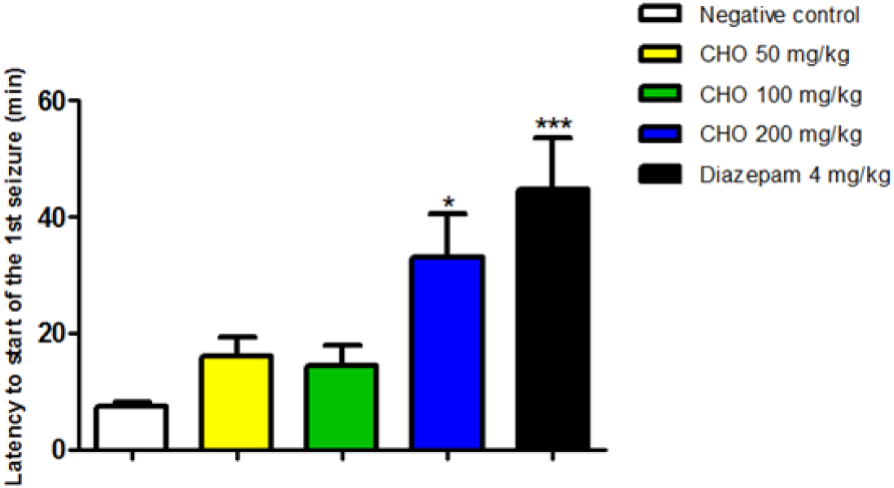
Effect of the essential oil of *Croton heliotropiifolius* Kunth (OCH), (*n* = 6/group). Leaves, at doses of 50, 100, and 200 mg/kg (v.o) on the latency to the first seizure induced by Pilorcapine (400 mg/kg, i.p.). Data are expressed as mean ± standard deviation.

Sesquiterpenes inhibit the enzyme GABA transaminase, leading to increased GABA levels in the central nervous system and resulting in sedative and tranquilizing effects.^52^ As shown in Figure 2, the groups treated with OCH (200 mg/kg) and diazepam exhibited a prolonged latency time before the onset of the first seizure, suggesting a mechanism of action similar to that of diazepam. Additionally, the latency to death was significantly extended in animals pretreated with OCH (200 mg/kg) (38.69 ± 19.97, **p < 0.01) and diazepam (60.00 ± 0.00, ***p < 0.001) compared to the negative control group (8.81 ± 2.18), the standard deviation is zero, as there were no deaths during the observation period (1 h). (Figure 3).

**Figure 3:**
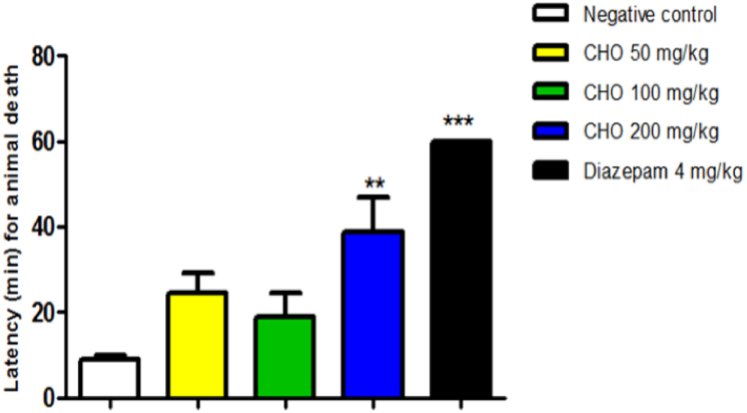
Effect of the essential oil of *Croton heliotropiifolius* Kunth. Leaves (OCH), at doses of 50, 100, and 200 mg/kg (v.o) on the latency to death of the animals, after the induction of convulsions by Pilocarpine (400 mg/kg, i.p.). Data are expressed as mean ± standard deviation (*n* = 6/group). Statistically significant difference if *p* < 0.05 (ANOVA followed by Tukey’s posttest), **p < 0.01 and ***p < 0.001, compared to the negative control group.

A study conducted with the essential oil of *Croton zehntneri* showed an increase in the threshold for the onset of minimal seizures induced by pentylenetetrazole (PTZ),^53^ suggesting the anticonvulsant potential of plants belonging to the genus *Croton*. The present study corroborates the results obtained with the OCH.

### 3.2 Molecular Docking

To understand the possible receptors and sites of action of the compounds in the oil, we carried out a molecular docking study focusing on the GABA_A_ and NMDA receptors. The accuracy of docking poses was estimated by redocking calculations. The RMSD values obtained for all the sites were less than 2 Å: site 1 (0.41 Å), site 2 (0.46 Å), site 3 (0.74 Å), site 4 (0.31 Å) and site 5 (0.62 Å) thus validating the method.^54^

The binding energy values obtained from the docking simulations were used to evaluate the affinity of the ligands for the GABA_A_ and NMDA receptors. These scores, which were calculated using AutoDock Vina, provide an empirical estimate of the binding energy.^55^ However, because this is a simplified model, the values obtained do not accurately reflect binding affinity. They are also limited by protein rigidity, the absence of an explicit solvent and entropically relevant simplifications. Therefore, the results should be considered a preliminary step in in silico screening.

#### 3.2.1 Docking with GABA_A_ Receptor

The GABA_A_ receptor is one of the main mediators of neuronal inhibition, acting in both synaptic and extrasynaptic regions to play a fundamental role in controlling brain excitability.^56^ Changes to this system can affect the pathophysiology of various types of epilepsy.

To investigate the binding affinity of the compounds for the GABA_A_, we performed docking studies. In molecular docking, the strength of the interaction between the ligands and the receptor is a measure of the binding energy; the lower the binding energy, the stronger the interaction.^57^ For the study of the affinity of the compounds for the target sites, it was considered that binding energies ≤ −7.0 kcal/mol indicate affinity with the receptors.^39^ Most of the compounds showed moderate to good binding energies for the target sites, with values ranging from −7.0 to −10.0 kcal/mol. The binding energies of all OCH phytoconstituents with the receptor are described in Table S2 (Supporting Information).

Among all the compounds analyzed, some ligands belonging to the sesquiterpene class stood out, showing good affinity for the BDZ binding sites, as shown in Table 1. In particular, the site 1 proved to be the most promising, with binding energies ranging from −9.1 to −10.0 kcal/mol, comparable to those obtained for diazepam (−10.3 kcal/mol) and clonazepam (−10.4 kcal/mol) drugs.

**Table 1:** Binding Energies Obtained for the Compounds with the Best Scores and Reference Drugs in the Target Binding Sites of the GABA_A_ Receptor

	Bond energy (kcal/mol)			
Compounds	GABA_A_			
	1	2	3	4
α-bulnesene	–10.0	–8.2	–8.7	–7.5
δ-cadinene	–9.7	–9.0	–8.8	–8.1
β-bourbonene	–9.2	–7.6	–8.0	–7.4
Guaiadiene	–9.1	–8.7	–8.7	–7.6
Diazepam	–10.3	–9.7	–9.5	–8.7
Clonazepam	–10.4	–9.0	–9.7	–8.6

As shown in Table 1, α-bulnesene exhibited the highest affinity for the GABA_A_ receptor (site 1), with a binding energy of −10.0 kcal/mol, followed by δ-cadinene, β-bourbonene and guaiadiene.

The sesquiterpenes germacrene D and B, β-elemene and epi-trans-caryophyllene exhibited moderate to strong affinity for site 1, with binding energies ranging from −7.7 to −9.1 kcal/mol (see Table S2). However, they exhibited lower affinity for the other sites, with binding energy values ranging from −5.9 to −8.3 kcal/mol. This behavior is similar to that observed for BDZs, which have a higher affinity for site 1 of the GABA_A_ receptor.^58^

In in vivo studies using pentylenetetrazol-induced seizure models, the monoterpenes limonene, myrcene and α-terpineol exhibited anticonvulsant effects, indicating a potential role as positive modulators of the GABA_A_. In our study, these molecules showed moderate affinity for BDZ sites (−7.0 to −7.6 kcal/mol).

Docking analysis of the monoterpenes α-pinene, β-pinene and sabinene revealed that these compounds have low affinity for the sites, with binding energies ranging from −5.9 to −6.9 kcal/mol. These in silico results corroborate with experimental data, suggesting that these molecules do not modulate the α1β2γ2 and α1β2 isoforms of GABA_A_.^56^

Our study found that the monoterpenes α-phellandrene, α-terpinolene and terpin-4-ol displayed a moderate affinity for site 1, ranging from −7.4 to −8.0 kcal/mol.

In addition to binding affinity, it is important to identify the nature of the intermolecular interactions present in the active site of the proteins. Figure 4 shows the interactions of the most promising ligands with the amino acid residues of the site 1, while the interactions with the other sites are shown in Figures S1–S3 (see Supporting Information).

**Figure 4:**
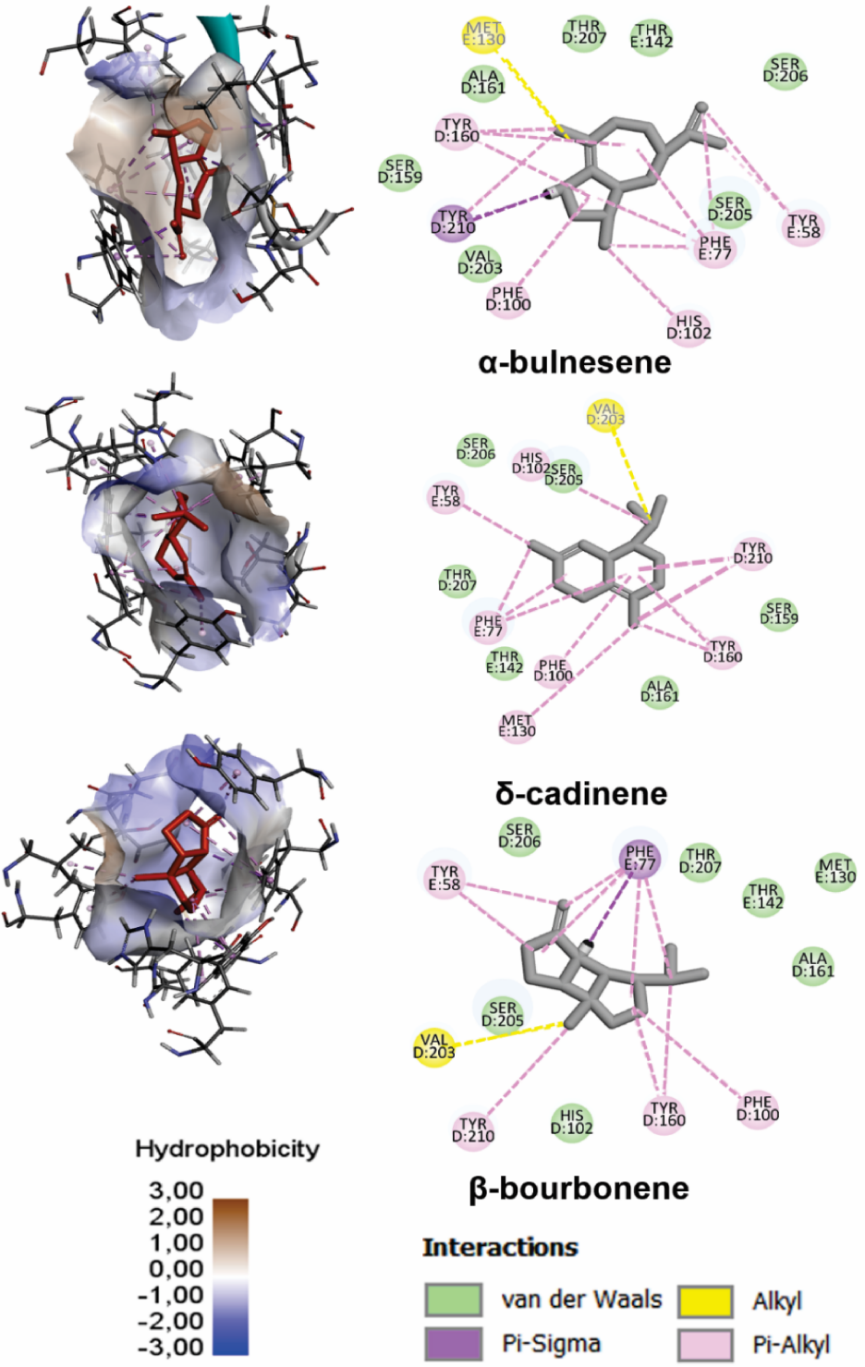
3D and 2D representations of ligands with the highest affinity for the α1+(D)/γ2^–^ (E) site (site 1) of the GABA_A_ receptor. The surface of the site is colored according to hydrophobicity (−3.0 to +3.0), with more hydrophobic regions in light/brown tones and less hydrophobic regions in blue. In the 3D images, the ligands occupy a predominantly hydrophobic pocket, formed mainly by Tyr, Phe, Val, and Met residues, which are also observed in the 2D representations, where alkyl and π–alkyl interactions predominate. Chains D and E correspond to the α1 and γ2 subunits, respectively.

Van der Waals, hydrophobic, electrostatic, and hydrogen bond interactions are the most common between ligands and proteins.^59^ Hydrophobic interactions occur between apolar groups and play a fundamental role in the protein–ligand docking process, since the active sites of proteins are predominantly composed of hydrophobic groups.^25,60^ As Figures 4 and S1–S3 show, ligands are primarily held at the BDZ sites of the GABA_A_ receptor by hydrophobic and π–alkyl interactions. At site 1, the γ2 subunit (E chain) significantly contributes to ligand stabilization. Notably, the Phe77 residue establishes multiple π–alkyl interactions with different portions of the ligands, as do the Met130 and Tyr58 residues. The α1 subunit (chain D) contributes to the formation of the binding pocket with residues Tyr210, Tyr160, Phe100, and His102.

The multiple interactions between the Phe77 in the γ2^–^(E) subunit and ligands suggest that this residue plays an important role in the affinity for the modulator site under investigation.

It was observed that the bicyclic structures of sesquiterpenes favor interactions with the amino acid Phe77, in contrast to the monoterpenes. These findings are consistent with experimental studies by Kessler et al.^56^ that suggest that the bicyclic nature of terpenes enhances modulation of the α1β2γ2 and α1β2 subunits of the GABA_A_ receptor.

#### 3.2.2 Docking with NMDA Receptor

NMDA glutamatergic receptors have been the target of clinical research, with evidence suggesting that a reduction of their activity can prevent seizures and neurodegeneration.^61^ Ifenprodil, a selective negative allosteric modulator of the GluN2B subunit of the NMDA receptor, has been shown to be more tolerable in animal models and humans than traditional NMDA antagonists, including high-affinity channel blockers, competitive antagonists and certain anticonvulsants.^62^ In this context, a docking study was conducted with the NMDA receptor at the binding site of ifenprodil.

Docking studies showed that the sesquiterpenes guaiadiene, δ-cadinene and α-bulnesene exhibited the best binding energies (−7.5 to −8.0 kcal/mol) at NMDA site 5, Table 2.

**Table 2:** Binding Energies Obtained for the Compounds with the Best Scores and Negative Allosteric Modulator in the Site 5 of the NMDA Receptor

Bond energy (kcal/mol)	
Compounds	NMDA
Guaiadiene	–8.0
α-bulnesene	–7.5
δ-cadinene	–7.5
α-terpinene	–7.5
ρ-cimene	–7.5
β-felandrene	–7.5
γ-terpinene	–7.5
Ifenprodil	–10.0

The binding energies of monoterpenes ranged from −4.9 to −7.5 kcal/mol. The compounds α-terpinene, ρ-cymene, β-phellandrene and γ-terpinene showed good affinity (−7.5 kcal/mol). In contrast, Oxygenated monoterpenes isoborneol and 1,8-cineole exhibited low affinity with binding energies of −5.0 and −5.3 kcal/mol, respectively Table S3 (see Supporting Information).

As shown in Figure 5, ligand binding to the NMDA receptor is primarily driven by hydrophobic interactions. The GluN1 subunit (chain C) stabilizes the binding pocket via residues Leu113, Phe91, and Tyr87. The GluN2B subunit (chain D) stabilizes the binding pocket via residues Ala466, Phe535, Ile470, and Pro437. Guaiadiene interacts with Leu113, Phe91, and Tyr87 (GluN1) and Ala466 and Phe535 (GluN2B). δ-Cadienene interacts with Leu113 (GluN1) and Ala466 and Phe535 (GluN2B). α-Bulnesene interacts with Leu113, Phe91, and Tyr87 (GluN1) and Ala466, Ile470, and Pro437 (GluN2B). These interactions contribute to the stabilization of the ligand-protein complexes.

**Figure 5:**
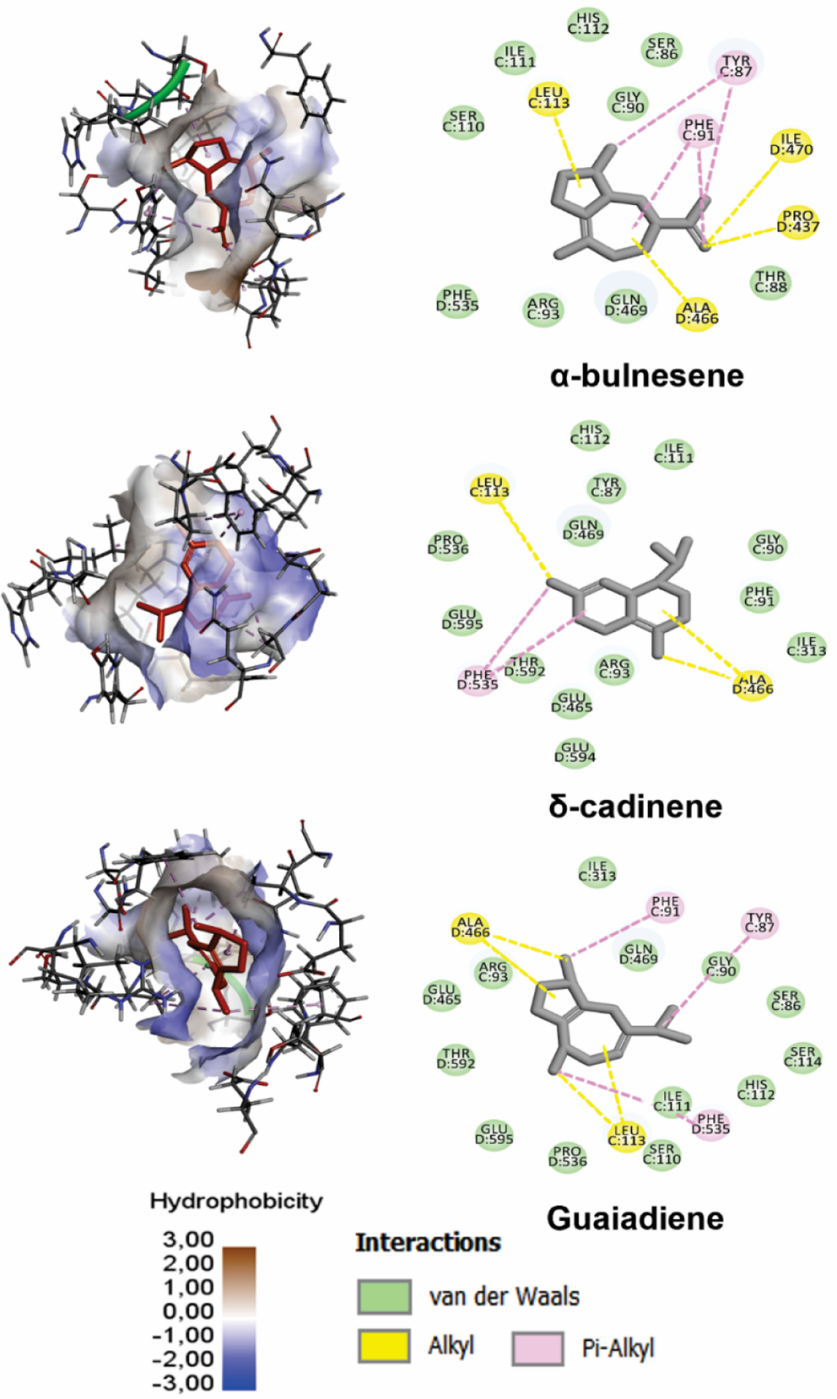
3D and 2D representations of ligands with the highest affinity for the GluN1­(C)-GluN2B­(D) site (site 5) of the NMDA receptor. The surface of the site is colored according to hydrophobicity (scale from −3.0 to +3.0), where more hydrophobic regions are represented in light and brown tones and less hydrophobic regions in blue tones. In the 3D images, the ligands are accommodated in a predominantly hydrophobic pocket, formed mainly by residues Phe91, Phe53, Try87, and Ala466. These same residues are identified in the 2D diagrams, in which alkyl and π–alkyl interactions predominate. Chains C and D correspond to the GluN1 and GluN2B subunits, respectively.

The observed interaction profile suggests that the apolar constituents of the plant could interact with NMDA, potentially acting as negative allosteric modulators.

### 3.3 DFT Calculation

#### 3.3.1 Molecular Orbital Analysis

The energies of the HOMO and LUMO frontier orbitals are often associated with the ability of a molecule to donate and accept electrons, respectively. The analysis of these orbitals and their energy gap is essential for predicting molecular reactivity and stability.^63^ Molecules with a large HOMO–LUMO gap tend to be less reactive, while those with a smaller gap exhibit higher reactivity. More reactive compounds are generally more likely to interact with biological targets,^64,65^ which may correlate with greater biological activity.^66^

Figure 6 shows the frontier molecular orbitals and their corresponding energy gaps, calculated at the B3LYP/6–311++G­(d,p)/SMD level, for the ligands with the highest affinity for GABA_A_ and NMDA receptors as identified in the docking study. The gap analysis suggests the following order of chemical reactivity: β-bourbonene < α-bulnesene ∼ δ-cadinene < guaiadiene.

**Figure 6:**
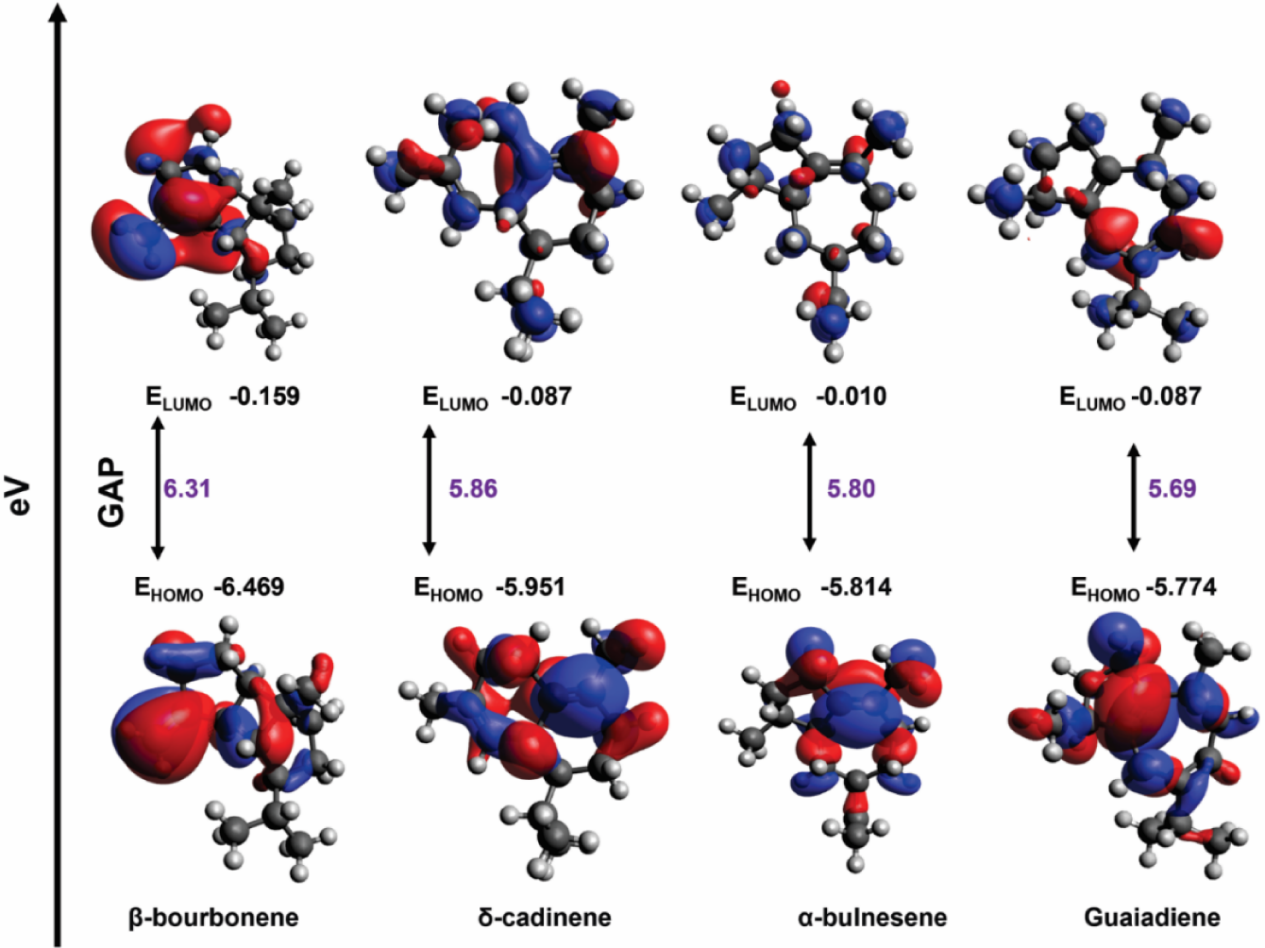
Frontier molecular orbitals and HOMO–LUMO energy gap of the compounds with the highest affinity for the receptors were calculated at the B3LYP/6–311++G­(d,p)/SMD level.

The plot of the frontier orbitals provides information about the nature of the molecular fragments associated with the electron donor/acceptor character. As shown in Figure 6, the HOMO orbitals show significant contributions from the π C=C bonds and conjugated regions of the molecules. It can be seen that the LUMO of α-bulnesene and δ-cadinene has the participation of the rings and unsaturated regions, while for the other molecules these orbitals are delocalized.

#### 3.3.2 Reactivity Descriptors

Conceptual DFT is an important tool for predicting the chemical reactivity of compounds and materials, as well as providing insight into the design and selection of potential therapeutic agents.^65,67^ The global chemical reactivity descriptors of the ligands with the highest affinity for the receptors were calculated and are shown in Table 3. The descriptors of all ligands are listed in Table S3 (see Supporting Information).

**Table 3:** Global Chemical Reactivity Descriptors of the Ligands with the Highest Affinities for the Receptors Calculated at the B3LYP/6–311++G­(d,p)/SMD Level in Water

	Chemical reactivity descriptors (eV)					
Compounds	(IP)	(EA)	(μ)	(η)	(χ)	(ω)
α-bulnesene	5.81	0.01	–2.91	2.90	2.91	1.46
δ-cadinene	5.95	0.09	–3.02	2.93	3.02	1.55
β-bourbonene	6.47	0.16	–3.31	3.16	3.31	1.74
Guaiadiene	5.77	0.09	–2.93	2.84	2.93	1.51

The ionization potential (IP) and electronic affinity (EA) indicate the ability of the molecule to release and accept electrons, respectively.^68^ The IPs of all compounds studied are higher than the EAs, indicating that the removal of an electron is energetically less favorable than its addition. β-Bourbonene had the highest ionization energy and electron affinity among the compounds, indicating its greater ability to accept electrons, which is consistent with its higher electronegativity.

Chemical hardness is a measure of a molecule’s resistance to the deformation of its electronic cloud under small perturbations during chemical processes.^69^ In general, molecules with high hardness are less polarizable and therefore more stable. In this context, β-bourbonene has the highest hardness and is the compound with the greatest chemical stability.

The electrophilicity index is a property associated with the ability of a chemical species to attract electrons from other species, indicating its electrophilic character.^66^ Molecules with an electrophilicity index below 0.8 eV are classified as weak electrophiles, those between 0.8 and 1.5 eV as moderate electrophiles, and those above 1.5 eV as strong electrophiles.^26,70^ Thus, as shown in Table 3, α-bulnesene, δ-cadinene and guaiadiene are considered moderate electrophiles, while β-bourbonene is classified as a strong electrophile.

In addition, the electrophilicity index may indicate overall toxicity, as toxicity tends to increase with increasing electrophilicity.^71^ Therefore, guaiadiene and α-bulnesene are expected to be less toxic than the other compounds.

In general, the compounds with the highest affinity for GABA_A_ and NMDA receptors are harder and have a moderate to strong electrophilic character compared to the molecules with lower affinity (isoborneol, α-pinene, β-pinene, and 1,8-cineol), see Table S3.

#### 3.3.3 Molecular Electrostatic Potential Surfaces

The molecular electrostatic potential (MEP) surface is a useful property for predicting the sites of electrophilic and nucleophilic reactivity of a molecule.^72^ The negative potential region (colored red or yellow) indicates the region with the highest electronic density and therefore the highest probability of electrophilic attack, while positive regions (colored blue) are more susceptible to nucleophilic addition. The green regions represent neutral sites. Analysis of the MEP surfaces, Figure 7, shows that the regions of electrophilic reactivity are located on the C=C bonds and the five- and six-membered rings, which may have favored the interaction with the Phe77 amino acid residues observed in the molecular docking study. While the positive regions are located in the CH_3_ and CH_2_ groups, which act as nucleophilic reactivity sites.

**Figure 7:**
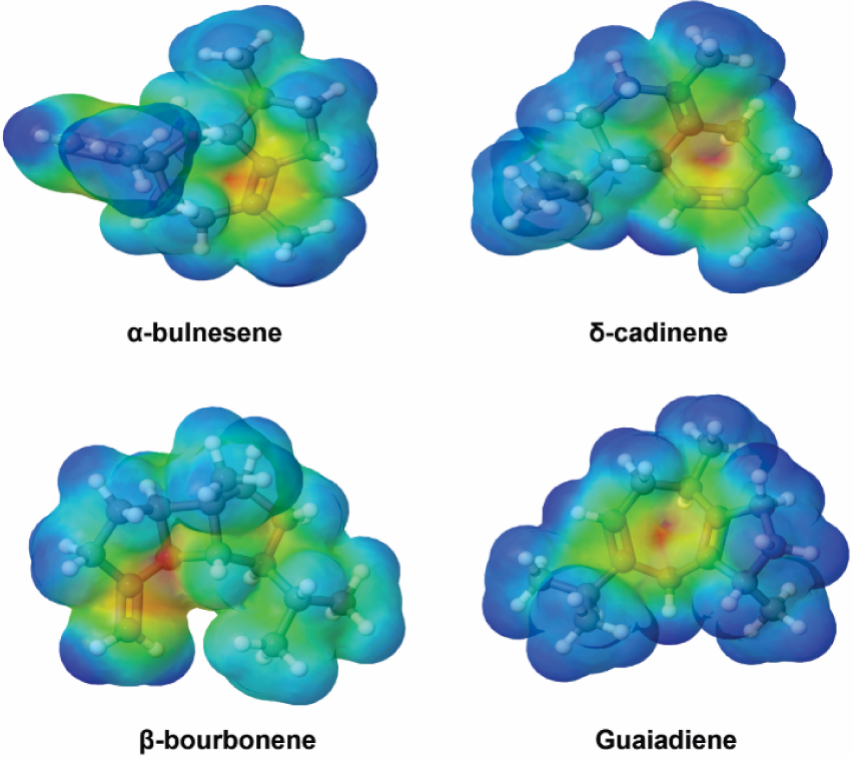
Molecular electrostatic potential maps of the compounds calculated at the B3LYP/6–311++G­(d,p) level with isosurface values of 0.002 au.

### 3.4 MD Simulations Analysis

Based on the docking results, the compounds α-bulnesene and guaiadiene, which have higher binding affinities for the GABA_A_ and NMDA receptors, respectively, were selected for molecular dynamics simulations.

Molecular dynamics analysis showed that both complexes reached stability after approximately 4 ns, remaining in their respective binding sites throughout the simulation period. The α-bulnesene–GABA_A_ complex showed high stability, with an average RMSD of 0.24 nm and a standard deviation of 0.10 nm Figure 8A. For the α-bulnesene ligand, the average RMSD was 0.09 nm with a standard deviation of 0.01 nm, indicating low structural fluctuation, 296 Col:751 Figure S4A.

**Figure 8:**
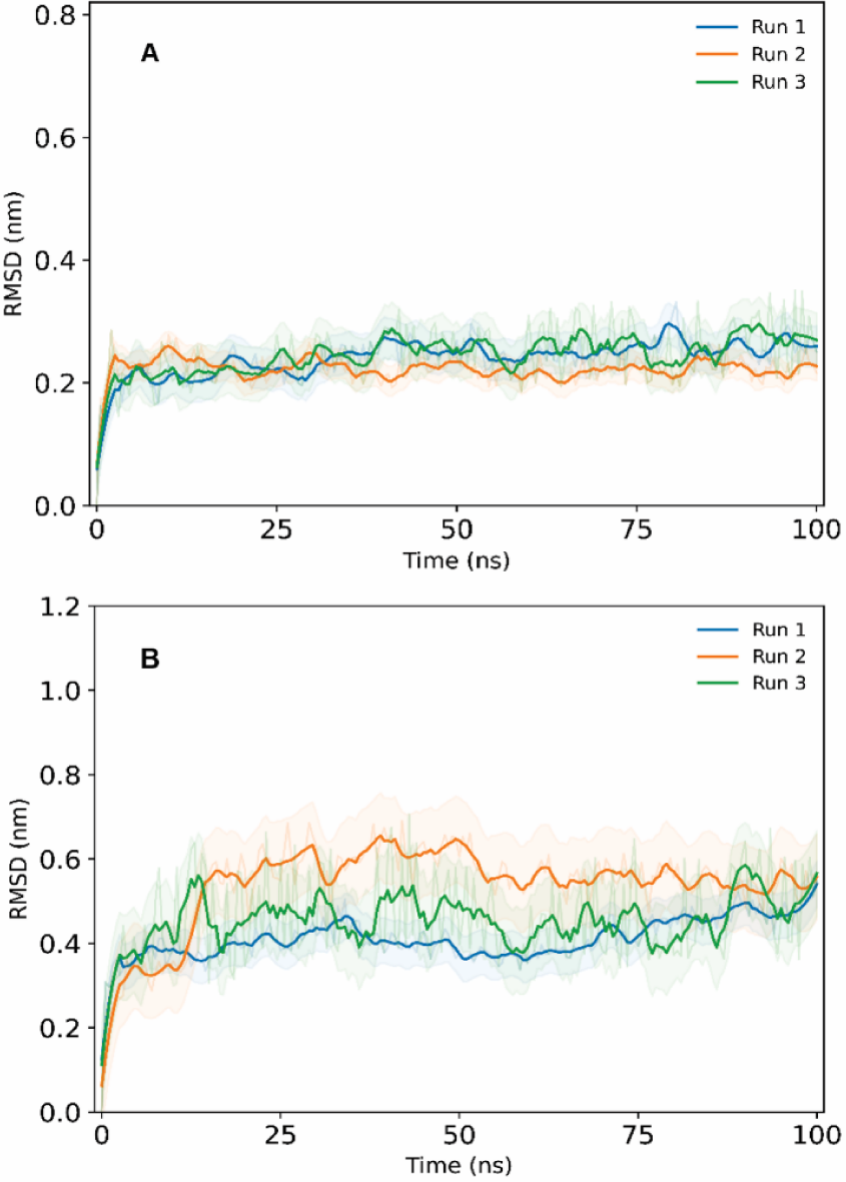
RMSD plots for (A) α-bulnesene–GABA_A_ complex and (B) guaiadiene–NMDA complex.

The guaiadiene–NMDA complex also demonstrated stability, with an average RMSD of 0.46 nm and a standard deviation of 0.10 nm (see Figure 8B). The guaiadiene ligand remained stable throughout the simulation, with an average RMSD of 0.05 nm and a standard deviation of 0.02 nm, Figure S4B It showed only minor variations in runs 1 and 3 after 75 ns, which are possibly related to reorientations or translations within the binding site. However, these fluctuations did not lead to dissociation, suggesting that they were only conformational adjustments to optimize interactions with site residues, maintaining a low average RMSD (<0.1 nm).

The RMSF values of the binding site residues, Figure S5 (see Supporting Information) reinforce this stability by showing low flexibility in these regions. Meanwhile, higher values are concentrated in the loops and terminal regions of the protein, as expected.^73^ The α-bulnesene–GABA_A_ and guanadiene–NMDA complexes had average radius of gyration values of 2.35 and 2.93 nm, respectively, with a standard deviation of 0.03 nm in both cases Figure S6 (see Supporting Information). This shows that they remained compact and structurally stable throughout the simulation.^74^

The average SASA values were 208.20 nm^2^ and 347.57 nm^2^ for the α-bulnesene–GABA_A_ and guaiadiene–NMDA complexes, respectively, with standard deviations of 3.83 nm^2^ and 5.78 nm^2^ Figure S7. These results suggest that the α-bulnesene–GABA_A_ complex has lower exposure to the solvent, which is consistent with greater stability and more robust interactions at the binding site.^75,76^

### 3.5 Binding Free Energy Analysis

The free energy of protein–ligand binding was calculated for the entire molecular dynamic’s trajectory using the molecular mechanics/Poisson–Boltzmann surface area (MM-PBSA) method, Figure 9. Based on the energy data, the guaiadiene–NMDA complex was found to be more stable, with a free binding energy of −22.22 kcal/mol (Figure 9B), compared to the α-bulnesene–GABA_A_ complex, which had a free binding energy of −15.29 kcal/mol (Figure 9A). This finding corroborates the RMSD data.^51,73^ The high van der Waals energy contributions of −27.22 kcal/mol (α-bulnesene–GABA_A_) and −32.14 kcal/mol (guaiadiene–NMDA) occur because these are nonpolar compounds, for which van der Waals interactions are the main contributor to stability.

**Figure 9:**
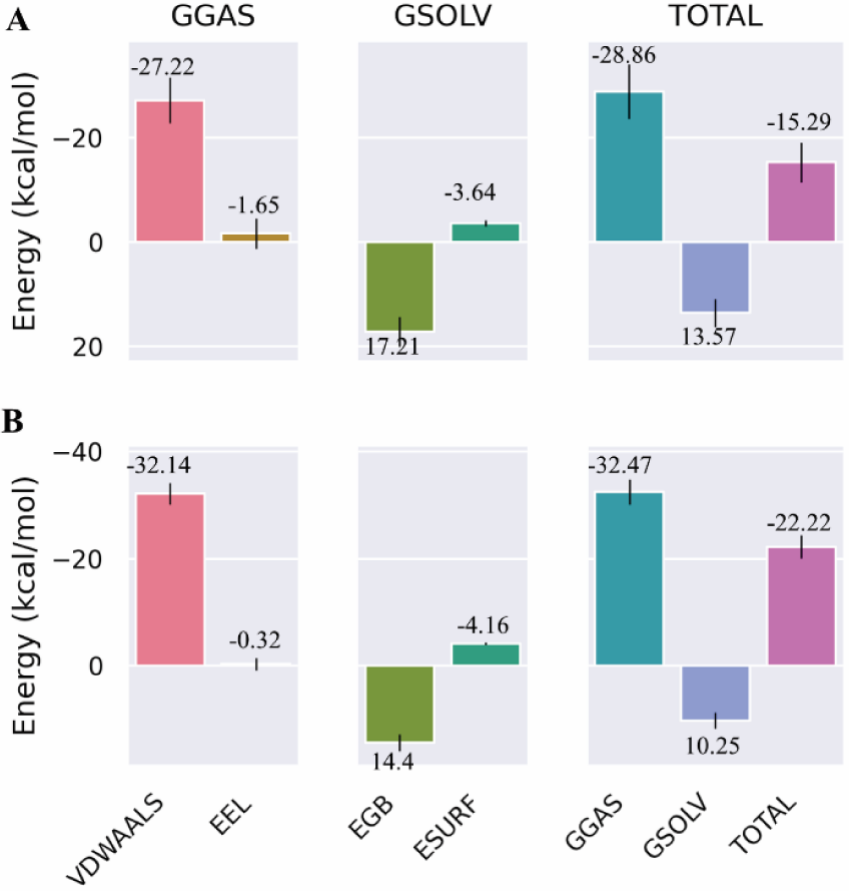
MM-PBSA analysis for (A) α-bulnesene–GABA_A_ complex and (B) guaiadiene–NMDA complex.

## 4 Conclusion

This study demonstrated for the first time the anticonvulsant properties of the essential oil of *C. heliotropiifolius* in a model using male mice. To corroborate the observed pharmacological activity, molecular docking studies of the constituents present in the oil with the GABA_A_ and NMDA receptors were performed. The docking simulations showed that the sesquiterpenes α-bulnesene, δ-cadinene, β-bourbonene and guaiadiene have high affinities for the BDZ sites on GABA_A_, possibly favored by their bicyclic structures. The ligand–receptor interactions were predominantly hydrophobic, highlighting the crucial role of the amino acid Phe77 present in the γ2-(E) subunit of GABA_A_. Docking analysis of NMDA revealed a higher affinity for the sesquiterpene guaiadiene. Molecular dynamics simulations showed that the α-bulnesene–GABA_A_ and guaiadiene–NMDA complexes remained stable throughout the 100 ns simulation. The energy profile indicates that these ligands have good affinity for the studied sites, corroborating the results of the molecular docking.

DFT studies indicated that the most promising ligands have high chemical stability and moderate to strong electrophilic character, which may contribute to the observed affinity. These findings broaden our understanding of how ligands interact with targets in the central nervous system, and could be used as a basis for developing new anticonvulsant agents. However, further studies are needed to assess the safety and clinical efficacy of the active components of the oil.

## Supplementary Material



## Data Availability

The data sets supporting this article have been uploaded as part of the electronic Supporting Information.

## Notes

Figure S1: 3D images of the ligands with the highest affinity for the β2+(C)/α1-(D) binding site (site 2) of GABA_A_; 2D images highlighting ligand–amino acid residue interactions at this site; Figure S2: 3D images of ligands with the highest affinity for the β2+(A)/α1-(B) binding site (site 3) of GABA_A_; 2D images highlighting ligand–amino acid residue interactions at this site; Figure S3: 3D images of ligands with the highest affinity for the β2-(A)/γ2+(E) binding site (site 4) of GABA_A_; 2D images highlighting ligand–amino acid residue interactions at this site; Figure S4: root mean square deviation (RMSD) of the ligands: (A) α-bulnesene, (B) guaiadiene; Figure S5: root mean square fluctuation (RMSF) of the complexes: (A) α-bulnesene–GABA_A_, (B) guaiadiene–NMDA; Figure S6: radius of gyration of the complexes: (A) α-bulnesene–GABA_A_, (B) guaiadiene–NMDA; Figure S7: solvent-accessible surface area (SASA) of complexes: (A) α-bulnesene–GABA_A_, (B) guaiadiene–NMDA. Table S1: protein residues involved in ligand interaction sites; coordinates of oxygen atoms used for site definition (*X*, *Y*, *Z*). Table S2: binding energies of OCH phytoconstituents at BDZ binding sites of the GABA_A_ receptor; Table S3: binding energies of OCH phytoconstituents at the NMDA receptor site; Table S4: global reactivity descriptors of OCH phytoconstituents obtained using B3LYP/6–311++G­(d,p)/SMD in water (PDF)
