# Supplementary material for: Anticonvulsant Potential of the Essential Oil of Croton Heliotropiifolius Kunth: In Vivo and In Silico Approach
Source: ACS Omega. 2026 Feb 4;11(6):8991–9002. doi: 10.1021/acsomega.5c04107 (PMC12917805; doi:10.1021/acsomega.5c04107)
Supplement: Supplementary file 1 [file ao5c04107_si_001.pdf]

Anticonvulsant potential of the essential oil of *Croton heliotropiifolius* Kunth: *In vivo* and *in silico* approach

Maria Elane S. da Cunha[□], Angélica L. Soares[§], Esdras M. S. Lima[§], Francisco A. S. Filho[#], Ricardo M. Ramos[⊥], Rosemarie B. Marques[∇], Francisco das C. P. de Andrade[τ], Anderson N. Mendes[τ,£], Evandro Paulo S. Martins[□,‡*]

[□]Postgraduate Program in Chemistry, State University of Piaui, Teresina, PI 64002-150, Brazil
[‡] State University of Piauí, Campus Antonio Giovanne Alves de Sousa, Piripiri, PI 64260-000, Brazil
[§]State University of Piauí, FACIME, Teresina, PI 64002-150, Brazil
[#] State University of Piauí, Campus Professor Alexandre Alves de Oliveira, Parnaíba, PI, Brazil
[⊥] Laboratory of Molecular Biology and Epidemiology (LABME), Federal Institute of Education, Science and Technology of Piauí, Teresina, PI 64002-150, Brazil
[∇] Biotechnology and Biodiversity Research Center, State University of Piauí, Teresina, PI 64002-150, Brazil
[τ] Laboratory of Innovation in Science and Technology – LACITEC, Department of Biophysics and Physiology, Federal University of Piauí, 64049-550, Teresina, Piauí, Brazil
[£] Department of Biophysics and Physiology, Federal University of Piauí, Teresina 64049-550, Brazil.

* Corresponding author:
Dr. Evandro Paulo Soares Martins
E-mail: evandropaulo@prp.uespi.br

Contents

FigureS1.3D images of the ligands with the highest affinity for the $\beta2^{+}$(C)/$\alpha1^{-}$(D) binding site (site 2) of $GABA_A$ and 2D images highlighting the interactions of the ligands with the amino acid residues in this site.

FigureS2. 3D images of the ligands with the highest affinity for the $\beta2^{+}$(A)/$\alpha1^{-}$(B) binding site (site 3) of $GABA_A$ and 2D images highlighting the interactions of the ligands with the amino acid residues in this site.

FigureS3.3D images of the ligands with the highest affinity for the $\beta2^{-}$(A)/$\gamma2^{+}$(E) binding site (site 4) of $GABA_A$ and 2D images highlighting the interactions of the ligands with the amino acid residues in this site.

FigureS4. Root mean square deviation (RMSD)of the ligands: (A) α-bulnesene, (B) Guaiadiene.

FigureS5. Root mean square fluctuation (RMSF) of the complexes:(A) α-bulnesene-$GABA_A$, (B) Guaiadiene-NMDA.

FigureS6. Radius gyration of the complexes: (A) α-bulnesene-$GABA_A$, (B) Guaiadiene-NMDA.

FigureS7. Solvent-accessible surface area (SASA) of the complexs: (A) α-bulnesene-$GABA_A$, (B) Guaiadiene-NMDA.

TableS1. Protein residues involved in the ligand interaction site and coordinates of the oxygen atoms used to define the site. (X, Y, Z).

TableS2.Binding energies of the OCH phytoconstituents with the BDZ binding sites on the $GABA_A$ receptor.

TableS3. Binding energies of OCH phytoconstituents with the NMDA receptor site.

TableS4. Global reactivity descriptors of the OCH phytoconstituents obtained by B3LYP/6-311++G(d,p)/SMD in water.

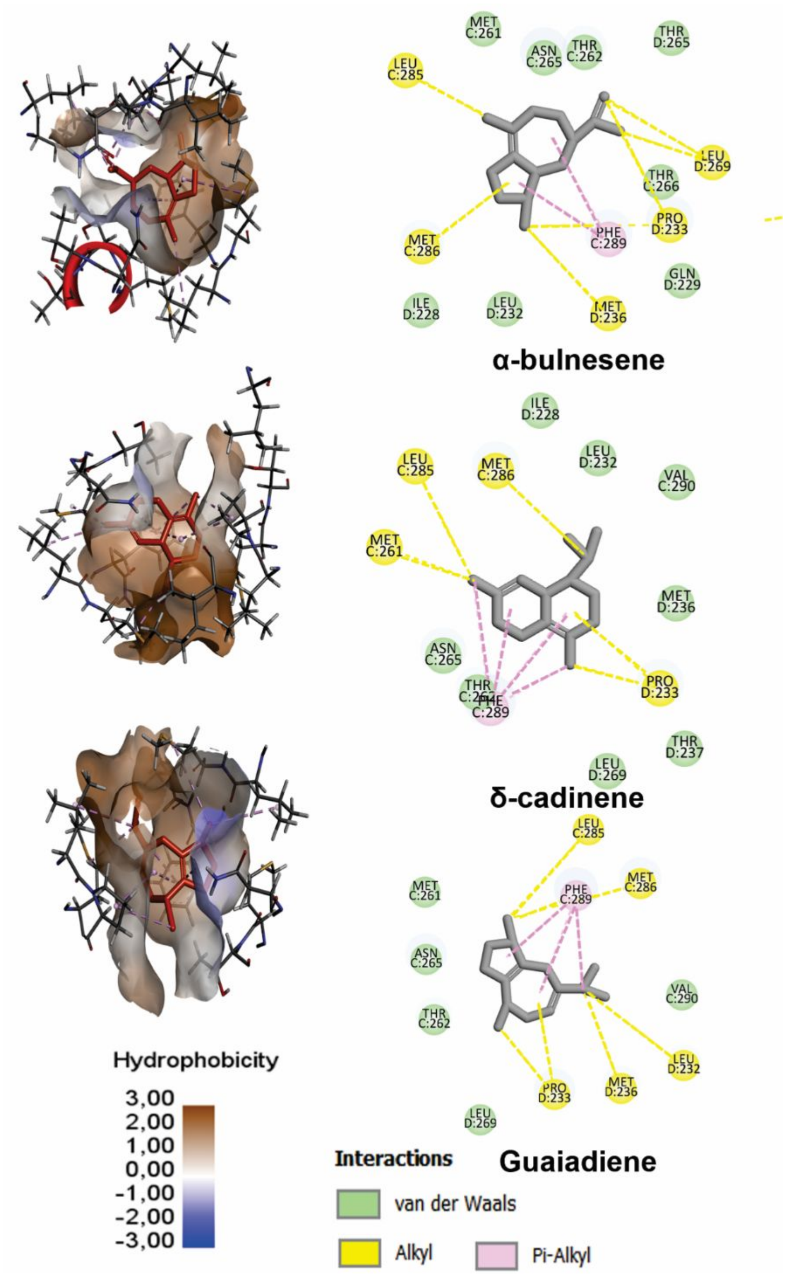

FigureS1.3D images of the ligands with the highest affinity for the β2⁺(C)/α1⁻(D) binding site (site 2) of $GABA_A$ and 2D images highlighting the interactions of the ligands with the amino acid residues in this site.

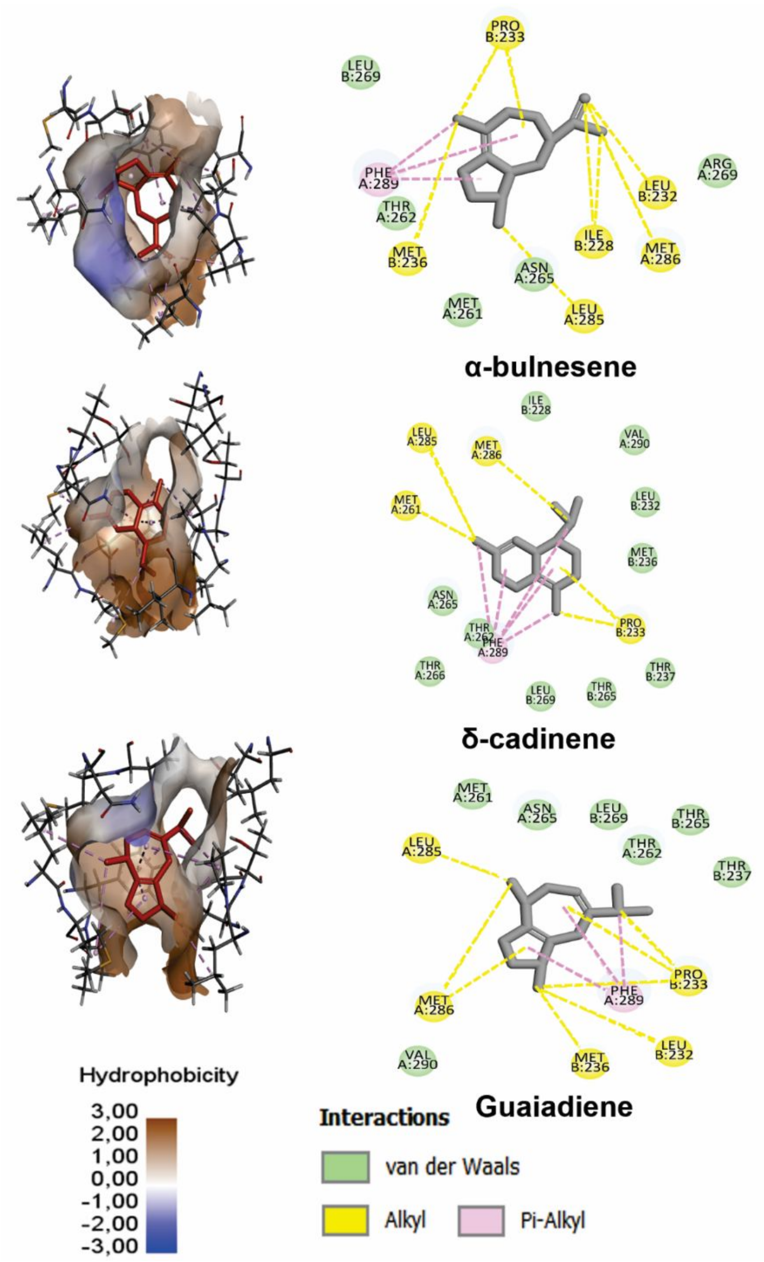

FigureS2. 3D images of the ligands with the highest affinity for the β2$^+$(A)/α1$^-$(B) binding site (site 3) of $GABA_A$ and 2D images highlighting the interactions of the ligands with the amino acid residues in this site.

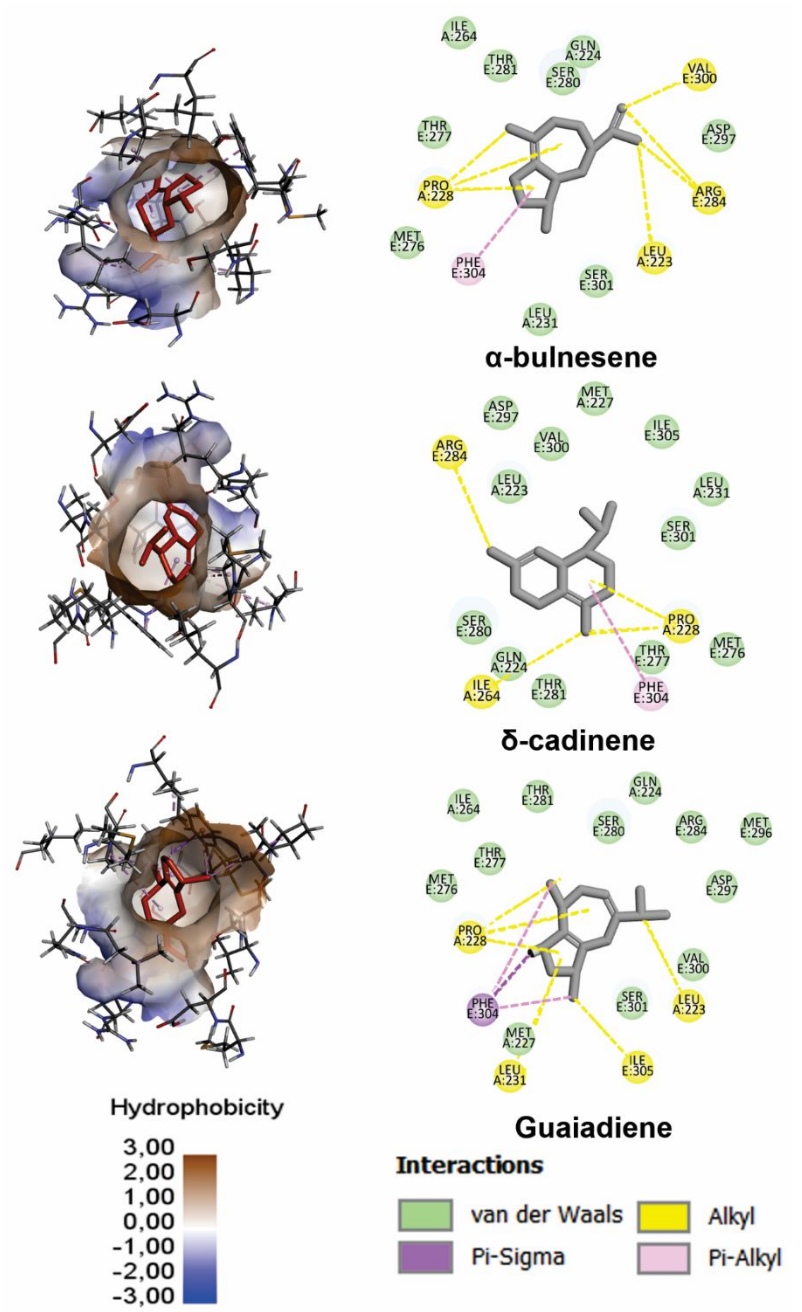

FigureS3.3D images of the ligands with the highest affinity for the $\beta2^{-}$(A)/$\gamma2^{+}$(E) binding site (site 4) of $GABA_A$ and 2D images highlighting the interactions of the ligands with the amino acid residues in this site.

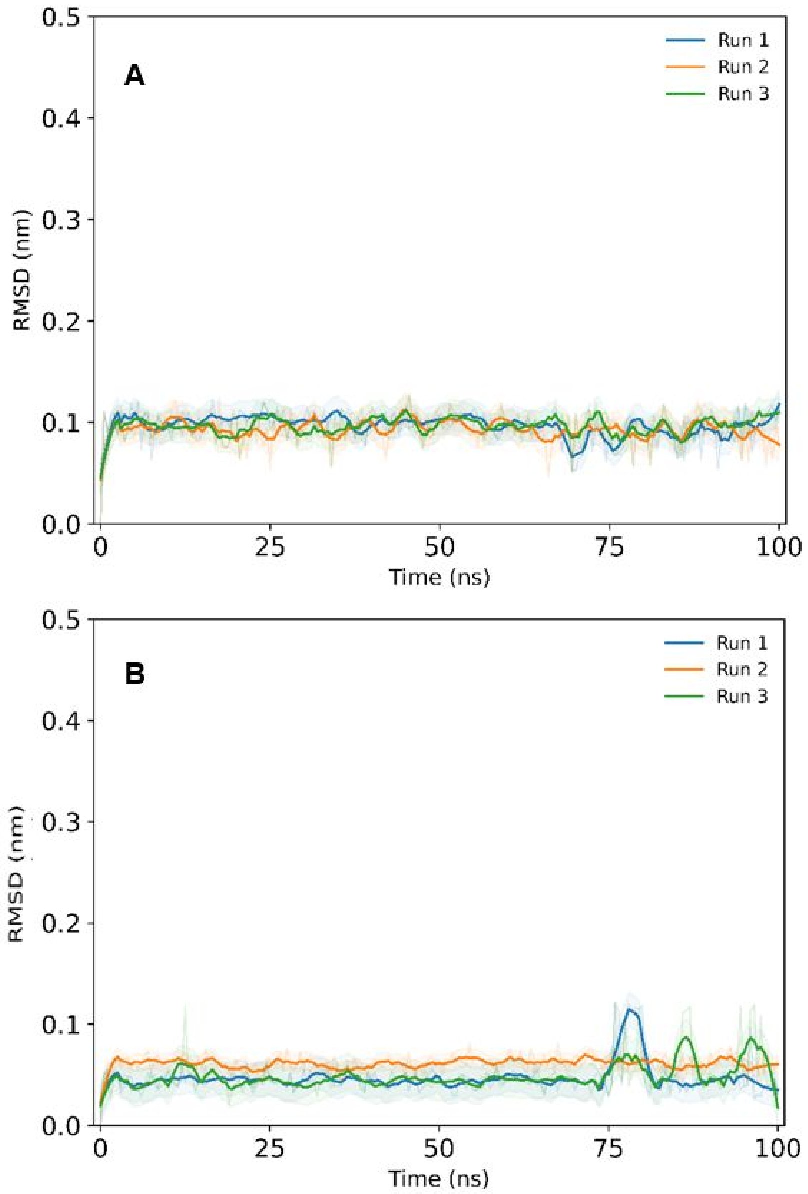

FigureS4. Root mean square deviation (RMSD)of the ligands: (A) α-bulnesene, (B) Guaiadiene.

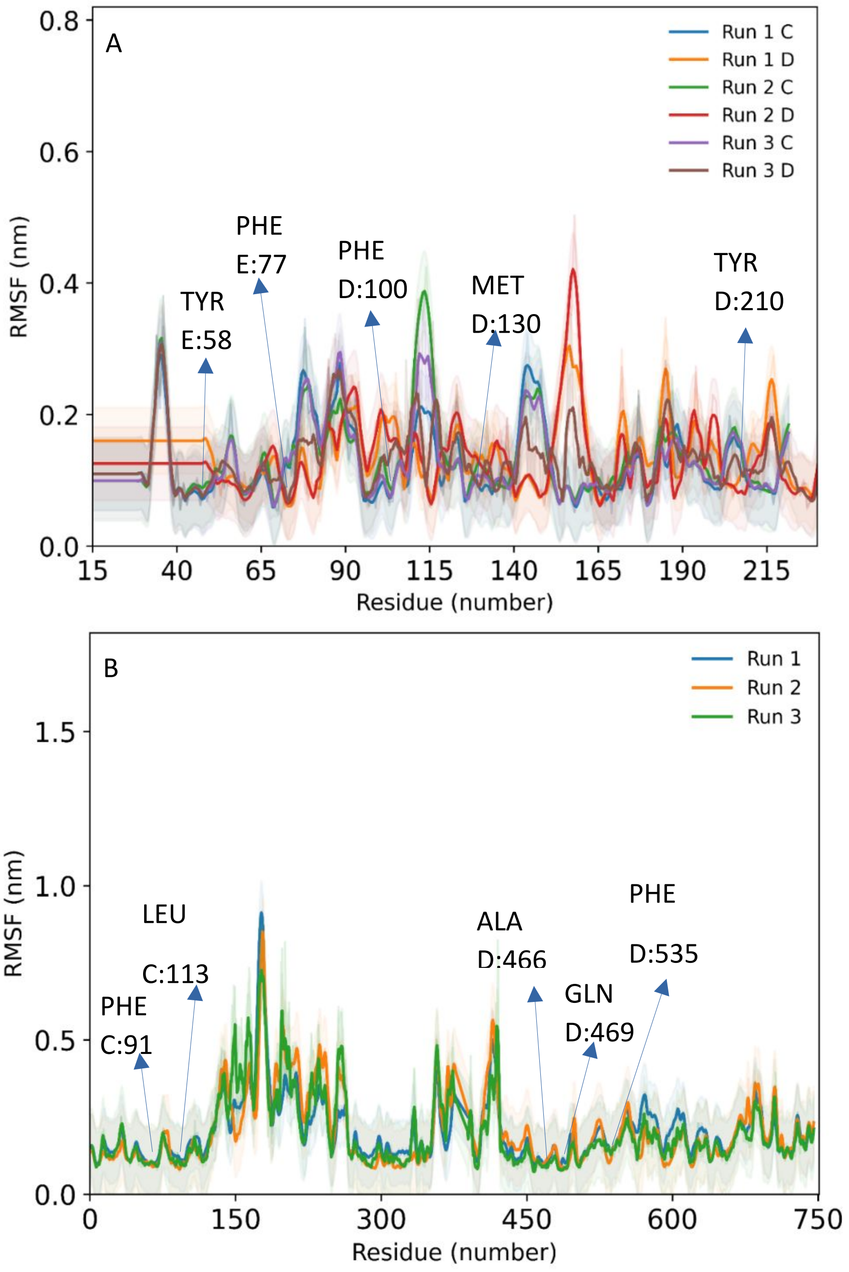

FigureS5. Root mean square fluctuation (RMSF) of the complexes: (A) α-bulnesene-$GABA_A$, (B) Guaiadiene-NMDA.

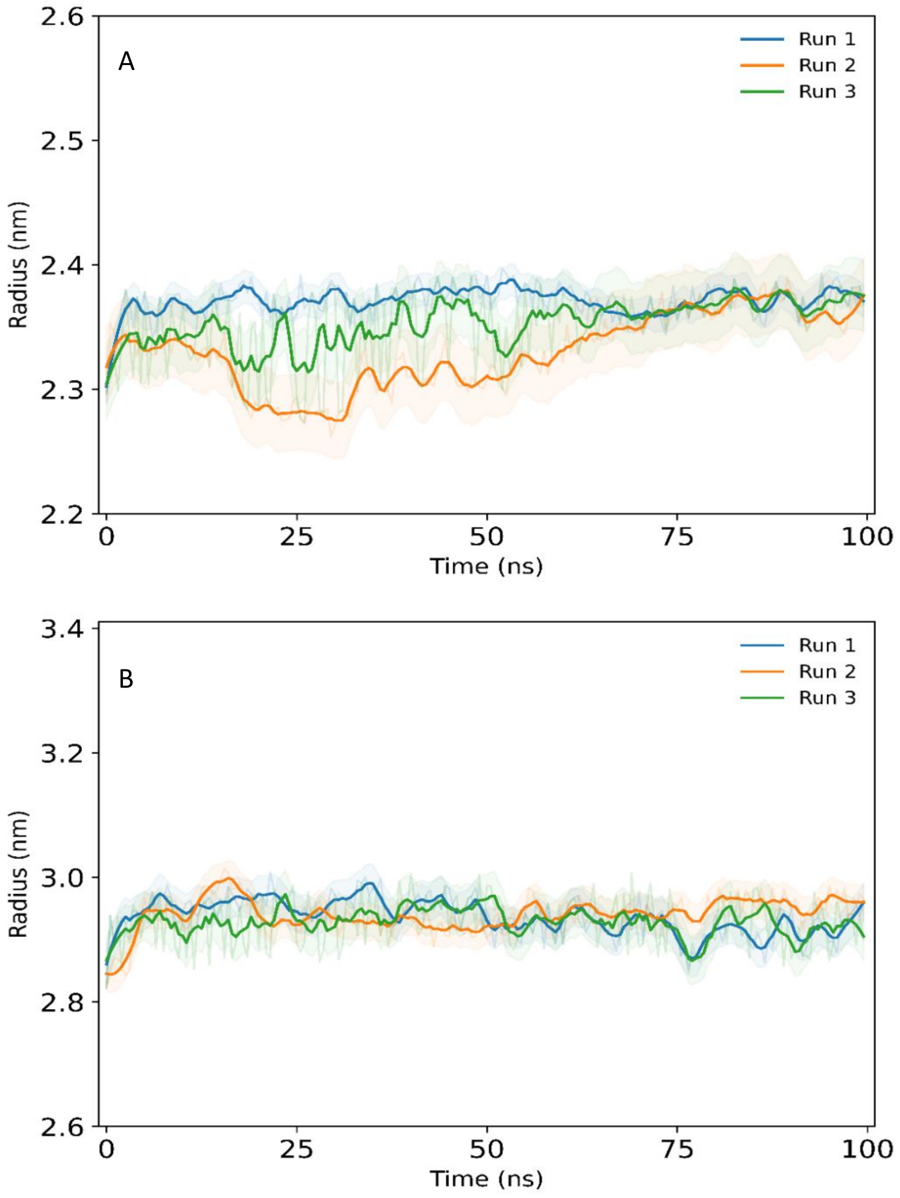

FigureS6. Radius gyration of the complexes: (A) α-bulnesene-$GABA_A$, (B) Guaiadiene-NMDA.

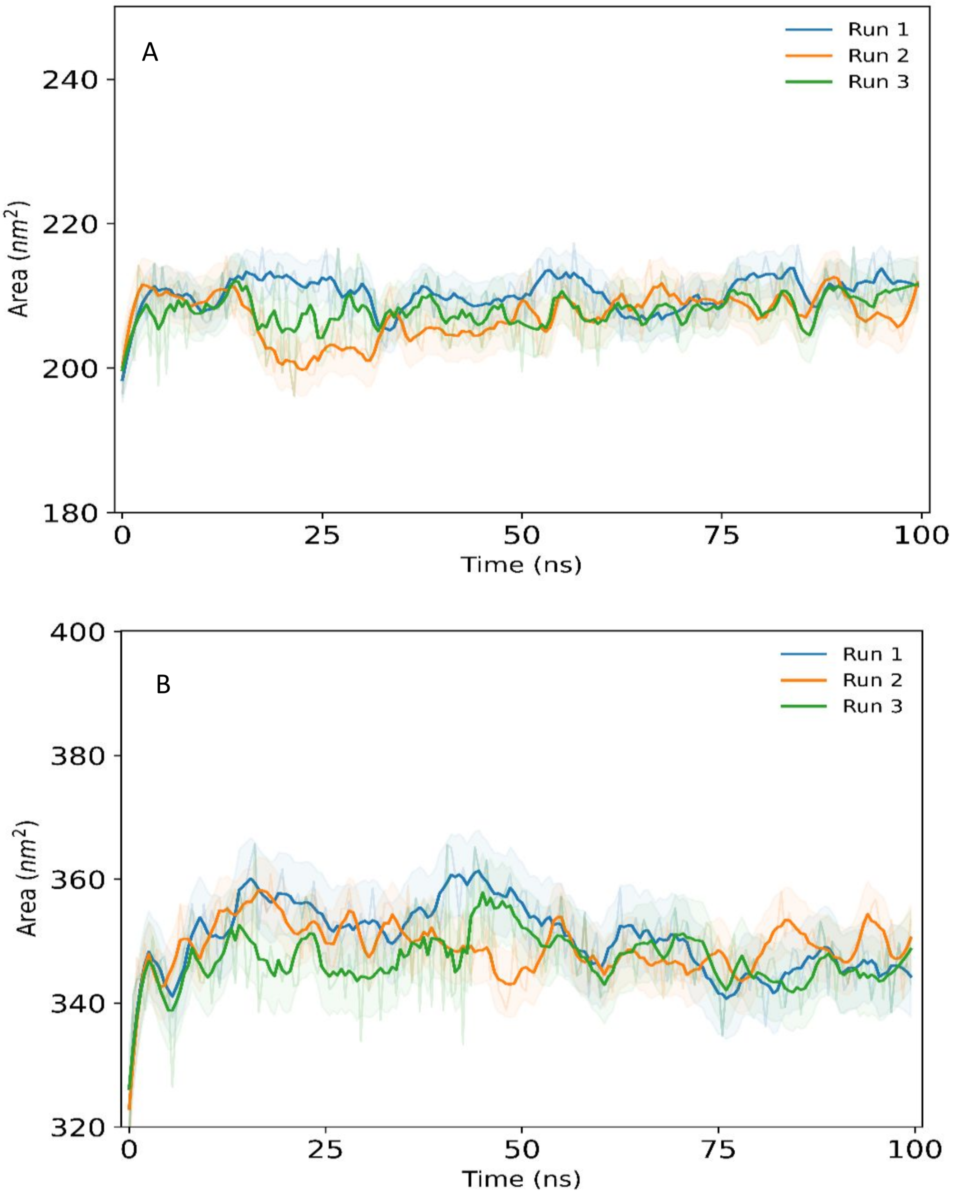

FigureS7. Solvent-accessible surface area (SASA) of the complexs: (A) α-bulnesene-$GABA_A$, (B) Guaiadiene-NMDA

TableS1. Protein residues involved in the ligand interaction site and coordinates of the oxygen atoms used to define the site. (X, Y, Z).

Site	Chain	Residue	Reference atom	Coordinates (x, y e z)
1	$\alpha1^{+}(D)/\gamma2^{-}(E)$	SER D:205	Oxygen	X=84.334, Y=128.228, Z=100.421
2	$\beta2^{+}(C)/\alpha1^{-}(D)$	ILED:228	Oxygen	X=120.919, Y=138.912, Z=133.941
3	$\beta2^{+}(A)/\alpha1^{-}(B)$	ILEB:228	Oxygen	X=143.346, Y=118.609, Z=123.685
4	$\beta2^{-}(A)/\gamma2^{-}(E)$	GLN A:224	Oxygen	X=137.534, Y=129.041, Z=110.812
5	GluN1/GluN2B	SER C:232	Oxygen	X=0.744, Y=29.422, Z=11.157

TableS2. Binding energies of the OCH phytoconstituents with the BDZ binding sites on the $GABA_A$ receptor.

	Bond energy (kcal/mol)			
Compounds	1	2	3	4
Monoterpene Hydrocarbons				
α-tujene	-7.4	-6.7	-6.4	-6.0
α -pinene	-6.4	-6.0	-6.0	-6.2
Sabinene	-6.9	-6.3	-6.2	-5.9
β- pinene	-6.3	-5.9	-5.9	-6.2
Myrcene	-7.0	-6.2	-5.8	-5.7
α-phellandrene	-7.4	-6.8	-6.7	-6.1
(+) - carene-3	-7.5	-6.6	-6.4	-6.4
α-terpinene	-7.4	-6.4	-6.5	-6.1
ρ-cimene	-7.4	-6.8	-6.8	-6.3
Limonene	-7.6	-6.7	-6.6	-6.1
β-phellandrene	-7.6	-6.7	-6.5	-6.3
ϒ-terpinene	-7.4	-6.7	-6.8	-6.3
α-terpinolene	-8.0	-7.1	-7.1	-6.1
Oxygenated Monoterpenes				
1,8-cineole	-6.2	-6.3	-6.2	-6.3
Linalool	-6.4	-6.0	-5.7	-6.3
Isoborneol	-5.7	-7.4	-6.2	-6.0
Terpin-4-ol	-7.7	-6.8	-6.8	-6.3
α-terpineol	-7.1	-6.6	-6.4	-6.5
Sesquiterpene Hydrocarbons				
β-bourbonene	-9.2	-7.6	-8.0	-7.4
β- elemene	-8.9	-7.8	-8.2	-6.8
β-isocumene	-5.7	-7.4	-7.4	-6.8
Trans-caryophyllene	-8.6	-7.2	-7.0	-6.9
Guaiadiene	-9.1	-8.7	-8.7	-7.6
α – humulene	-7.8	-6.6	-7.0	-5.7
Epi-trans- caryophyllene	-8.1	-7.9	-7.9	-6.9
Germacrene-D	-9.1	-8.2	-8.3	-6.5
Biciclogemacrene	-6.8	-8.5	-8.3	-7.1

α-bulnesene	-10.0	-8.2	-8.7	-7.5
δ-cadinene	-9.7	-9.0	-8.8	-8.1
Oxygenated Sesquiterpenes				
Germacrene-B	-7.7	-7.2	-7.2	-5.9
Spatulenol	-8.8	-8.3	-8.1	-6.5
caryophyllene oxide	-7.3	-7.7	-7.9	-6.0
Isospathulenol	-9.2	-6.9	-7.0	-7.7
Reference drugs				
Diazepam	-10.3	-9.7	-9.5	-8.7
Clonazepam	-10.4	-9.0	-9.7	-8.6

TableS3. Binding energies of OCH phytoconstituents with the NMDA receptor site.

Compounds	Bond energy (kcal/mol)
	Site 5
Monoterpene Hydrocarbons	
α-tujene	-7.0
α -pinene	-6.9
Sabinene	-6.6
β- pinene	-7.1
Myrcene	-6.6
α-phellandrene	-7.4
(+) - carene-3	-6.4
α-terpinene	-7.5
ρ-cimene	-7.5
Limonene	-7.4
β-phellandrene	-7.5
ϒ-terpinene	-7.5
α-terpinolene	-6.5
Oxygenated Monoterpenes	
1,8-cineole	-5.3
Linalool	-6.5
Isoborneol	-5.0
Terpin-4-ol	-6.5
α-terpineol	-7.3
Sesquiterpene Hydrocarbons	
β-bourbonene	-6.9
β- elemene	-6.6
β-isocumene	-6.4
Trans-caryophyllene	-6.5
Guaiadiene	-8.0
α – humulene	-6.3
Epi-trans- caryophyllene	-7.0
Germacrene-D	-7.0
Biciclogemacrene	-6.1
α-bulnesene	-7.5
δ-cadinene	-7.5
Oxygenated Sesquiterpenes	
Germacrene-B	-6.7
Spatulenol	-7.4
caryophyllene oxide	-6.4
Isospathulenol	-6.9
Reference inhibitor	
ifenprodil	-10.0

TableS4. Global reactivity descriptors of the OCH phytoconstituents obtained by B3LYP/6-311++G(d,p)/SMD in water.

Compounds	Chemical reactivity descriptors (eV)						
	(IP)	(EA)	(μ)	(η)	(χ)	(ω)	GAP (eV)
Monoterpene Hydrocarbons							
α-tujene	5.946	-0.024	-2.961	2.985	2.961	1.469	5.97
α-pinene	6.002	-0.013	-2.995	3.008	2.995	1.491	6.02
Sabinene	6.216	-0.013	-3.102	3.115	3.102	1.544	6.23
β-pinene	6.396	-0.019	-3.189	3.208	3.189	1.585	6.42
Myrcene	6.261	0.952	-3.607	2.655	3.607	2.450	5.31
α-phellandrene	5.696	0.696	-3.196	2.500	3.196	2.043	5.00
(+) -carene-3	6.194	0.060	-3.127	3.067	3.127	1.594	6.13
α-terpinene	5.411	0.562	-2.987	2.425	2.987	1.839	4.85
ρ-cimene	6.353	0.295	-3.324	3.029	3.324	1.824	6.06
Limonene	6.231	0.021	-3.126	3.105	3.126	1.574	6.21
β-felandrene	6.044	0.760	-3.402	2.642	3.402	2.190	5.28
ϒ terpinene	5.995	0.052	-3.024	2.972	3.024	1.538	5.94
α-terpinolene	5.979	0.090	-3.035	2.945	3.035	1.564	5.89
Oxygenated Monoterpenes							
1.8-cineole	6.586	0.025	-3.306	3.281	3.306	1.665	6.56
Linalool	6.280	0.103	-3.192	3.089	3.192	1.649	6.18
Isoborneol	7.251	0.048	-3.650	3.602	3.650	1.849	7.30
Terpin-4-ol	6.322	0.108	-3.215	3.107	3.215	1.663	6.21
α-terpineol	6.211	0.061	-3.136	3.075	3.136	1.599	6.15
Sesquiterpene Hydrocarbons							
β-bourbonene	6.469	0.159	-3.314	3.155	3.314	1.741	6.31
β-elemene	6.478	0.038	-3.258	3.220	3.258	1.648	6.44
β-isocumene	6.631	0.320	-3.476	3.156	3.476	1.914	6.31
Trans-caryophyllene	6.018	0.063	-3.041	2.978	3.041	1.552	5.96
Guaiadiene	5.774	0.087	-2.931	2.844	2.931	1.510	5.69
α-humulene	5.957	0.007	-2.982	2.975	2.982	1.495	5.95
Epi-trans-caryophyllene	6.047	0.103	-3.075	2.972	3.075	1.591	5.94
Germacrene-D	5.552	0.531	-3.042	2.511	3.042	1.842	5.02
Biciclogemacrene	5.513	0.031	-2.772	2.741	2.772	1.402	5.48
α-bulnesene	5.814	0.010	-2.912	2.902	2.912	1.461	5.80
δ-cadinene	5.951	0.087	-3.019	2.932	3.019	1.554	5.86
Oxygenated Sesquiterpenes							
Germacrene-B	5.732	0.046	-2.889	2.843	2.889	1.468	5.69
Spatulenol	6.354	0.072	-3.213	3.141	3.213	1.643	6.28
caryophyllene oxido	6.447	0.049	-3.248	3.199	3.248	1.649	6.40
Isospathulenol	5.857	0.071	-2.964	2.893	2.964	1.518	5.79
